# Biodiversity checklists for Bhutan

**DOI:** 10.3897/BDJ.10.e83798

**Published:** 2022-05-19

**Authors:** Choki Gyeltshen, Karunya Prasad

**Affiliations:** 1 National Biodiversity Centre, Thimphu, Bhutan National Biodiversity Centre Thimphu Bhutan

**Keywords:** checklist, Bhutan, plants, animals, insects, vertebrates, mammals, birds, reptiles, amphibians, fish, invertebrates, fungi, chromista, eubacteria, protista, biodiversity

## Abstract

**Background:**

The species checklists for Bhutan published in the Global Biodiversity Information Facility (GBIF) were originally collated for the publication ‘Biodiversity Statistics of Bhutan 2017, A Preliminary Baseline’ (BSB 2017). This document was published in 2019 and is the first comprehensive overview of Bhutan's species richness, recording more than 11,000 species across all five kingdoms. Collation of biodiversity checklists has been limited in Bhutan. Thus, this paper and its associated data provide an overview of all species from the kingdoms Plantae, Animalia, Fungi, Chromista and Eubacteria in the country that have been recorded in scientific publications.

**New information:**

The checklists showcase 11,175 species from the kingdoms Plantae, Animalia, Fungi, Chromista and Eubacteria. Research conducted into identifying species from the kingdoms Protista and Archaebacteria revealed zero records. These checklists include at least 33 species new to science and 566 species new to Bhutan, discovered between 2009 and 2017. Unidentified species are not taken into account in this publication.

## Introduction

The Himalayan country of Bhutan is renowned for its rich biodiversity (Table 1) consisting of endemic, native and endangered flora and fauna which inhabit ecosystems including tropical/subtropical/temperate forests, tropical/subtropical grasslands, alpine meadows and scree. A total of 70.46% of the nation's land is protected habitat and the elevation ranges from 100 metres above sea level (masl) to 7,570 masl at the peak of Gangkhar Phuensum. The International Union for Conservation of Nature (IUCN) Red List has categorised 131 species from the kingdoms Animalia and Plantae as being threatened with extinction: 24 species are critically endangered, 47 species endangered and 60 species vulnerable (IUCN 2021).

While the biodiversity found in these diverse habitats has been recorded both formally and informally over time, the collaboration of stakeholders interested in the conservation of these species and collation of related data have been limited. Therefore, in December 2016, the National Biodiversity Centre (NBC) in Thimphu, Bhutan, began the coordination and gathering of species checklists from various taxonomic experts and specialist institutions in Bhutan. The taxonomic data were discussed over multiple workshops before being finalised by a range of contributors from government agencies, universities, local and international non-government organisations, as well as other private institutions and colleges. Comprehensive literature reviews were conducted to collect as much information as possible to develop the checklists.

## General description

### Purpose

The checklists were created to provide biodiversity statistics baselines for Bhutan. These species checklists can now be updated regularly with new species records and discoveries made within the country. As the nation's first comprehensive biodiversity list, these data deliver a reliable reference point for Bhutan's species, available for use by scientists, researchers, civil servants and the general public.

## Project description

### Title

Biodiversity checklists for Bhutan

### Personnel

National Biodiversity Centre

### Study area description

Bhutan - eastern Himalayas

### Design description

Biodiversity data were obtained from various sources and a national biodiversity checklist was developed and published in 2019 as the BSB 2017, by the National Biodiversity Centre (National Biodiversity Centre (NBC) 2019). This checklist has been divided into categories taxonomically and all species have been registered with GBIF. The Plantae and Animalia kingdoms account for a majority 93.2% of the total number of species found in Bhutan (Table 2).

### Funding

BIFA - GBIF (BIFA5_008)

## Sampling methods

### Study extent

Bhutan

### Sampling description

Data sources include: NBC; Sherubtse College; College of Natural Resources; Naturalis Biodiversity Centre (The Netherlands); Ugyen Wangchuck Institute for Conservation and Environmental Research; Department of Forests and Park Services; Department of Livestock; National Mushroom Centre; National Plant Protection Centre; National Herbarium and the National Invertebrate Repository Centre at the NBC. Comprehensive literature reviews were conducted in order to access all available scientific publications containing information on species records.

### Quality control

Data were validated using published resources, such as peer-reviewed journals, books, institutional databases, theses and museum specimens located at the NBC and other national taxonomic agencies. Online databases, such as the Catalogue of Life, IUCN, BirdLife, GBIF and Australian Plant Name Index, were also used for nomenclature and taxonomic classification verification.

Species records from media sources, such as newspapers, magazines, television and social media, as well as anecdotal and non-scientific sources, were not included. Additionally, a lack of access to paid scientific publications and limited publishing of new records in peer-reviewed journals and other scientifically-robust modes of communication by local researchers could have led to some new records and discoveries being overlooked. Unidentified species have also not been taken into account.

### Step description

1. Data collected from various sources, literature reviews conducted and draft checklists produced.
2. Cross-checked and conducted quality control of draft checklists against validation resources.
3. Two national stakeholder workshops conducted with attendees from all relevant agencies and institutions across Bhutan gathering to discuss the first and second drafts of the checklists. Relevant experts who were unable to attend the workshops were also requested to review the checklists before they were finalised.
4. BSB 2017 (National Biodiversity Centre (NBC) 2019) published.
5. Registered checklists (with minor revisions) with GBIF.

## Geographic coverage

### Description

Bhutan, entire country

### Coordinates

 and 27°31'53.11" Latitude; and 90°26'9.07" Longitude.

## Taxonomic coverage

### Taxa included

**Table:** 

Rank	Scientific Name	
kingdom	Animalia	
kingdom	Plantae	
kingdom	Fungi	
kingdom	Chromista	
kingdom	Eubacteria	

## Temporal coverage

**Formation period:** Data collected until end 2017.

## Usage licence

### Usage licence

Creative Commons Public Domain Waiver (CC-Zero)

## Data resources

### Data package title

Biodiversity Checklists for Bhutan

### Number of data sets

6

### Data set 1.

#### Data set name

Bhutan: Chromista & Eubacteria species

#### Download URL

https://doi.org/10.15468/rsvcsf; https://cloud.gbif.org/bifa/resource?r=bhutan_chromista_eubacteria

#### Description

A total of 75 records including diatom, brown algae, cyanobacteria, bacteria and protozoa species (Gyeltshen and Prasad 2022b).

**Data set 1.** 

Column label	Column description
Kingdom	Kingdom
Phylum	Phylum
Class	Class
Order	Order
Family	Family
Scientific Name	Species / subspecies / variety name
Vernacular Name	Common name
Scientific Name Authorship	Scientific name authorship

### Data set 2.

#### Data set name

Bhutan: Fungi species

#### Download URL

https://doi.org/10.15468/x49n78; https://cloud.gbif.org/bifa/resource?r=bhutan_fungi

#### Description

A total of 690 records including insect-fungi, Basidiomycota, Ascomycota, Oomycota and lichen species (Gyeltshen and Prasad 2022a).

**Data set 2.** 

Column label	Column description
Kingdom	Kingdom
Phylum	Phylum
Class	Class
Order	Order
Family	Family
Scientific Name	Species / variety name
Vernacular Name	Common name
Scientific Name Authorship	Scientific name authorship

### Data set 3.

#### Data set name

Bhutan: Invertebrate species

#### Download URL

https://doi.org/10.15468/rjuepc; https://cloud.gbif.org/bifa/resource?r=bhutan_invertebrates

#### Description

A total of 3,917 records including crab, spider, Psocoptera, mite, snail, slug and nematode species (Gyeltshen and Prasad 2022c).

**Data set 3.** 

Column label	Column description
Kingdom	Kingdom
Phylum	Phylum
Class	Class
Order	Order
Scientific Name	Family / genus / species / subspecies name

### Data set 4.

#### Data set name

Bhutan: Nonvascular plant species

#### Download URL

https://doi.org/10.15468/kv2xcp; https://cloud.gbif.org/bifa/resource?r=nonvascular_plants

#### Description

A total of 801 records including fern, liverwort, hornwort, moss and algae species (Gyeltshen and Prasad 2022f).

**Data set 4.** 

Column label	Column description
Kingdom	Kingdom
Phylum	Phylum
Class	Class
Order	Order
Family	Family
Scientific Name	Genus / species / subspecies / variety name
Scientific Name Authorship	Scientific name authorship

### Data set 5.

#### Data set name

Bhutan: Vascular plant species

#### Download URL

https://doi.org/10.15468/p82afy; https://cloud.gbif.org/bifa/resource?r=v_plants

#### Description

A total of 4,546 records of seed plant species (Gyeltshen and Prasad 2022e).

**Data set 5.** 

Column label	Column description
Kingdom	Kingdom
Phylum	Phylum
Class	Class
Order	Order
Family	Family
Scientific Name	Genus / species / subspecies / variety name

### Data set 6.

#### Data set name

Bhutan: Vertebrate species

#### Download URL

https://doi.org/10.15468/n6jv4s; https://cloud.gbif.org/bifa/resource?r=bhutan_vertebrates

#### Description

A total of 1,146 records including mammal, bird, amphibian, reptile and fish species (Gyeltshen and Prasad 2022d).

**Data set 6.** 

Column label	Column description
Kingdom	Kingdom
Phylum	Phylum
Class	Class
Scientific Name	Species / subspecies name

## Figures and Tables

**Table 1.** Number of species in Bhutan compared to the world (Mora et al. 2011).

Kingdom	World	Bhutan	Bhutan %
Animalia	1124516	5063	0.45
Plantae	224244	5347	2.38
Fungi	44368	690	1.56
Chromista	17892	57	0.32
Protista	16236	0	0.00
Eubacteria	11010	18	0.16
Archaebacteria	503	0	0.00

**Table 2.** Number and percentage of species by kingdom.

Kingdom	Number	Percentage
Animalia	5063	45.31
Plantae	5347	47.85
Fungi	690	6.17
Chromista	57	0.51
Protista	0	0.00
Eubacteria	18	0.16
Archaebacteria	0	0.00
